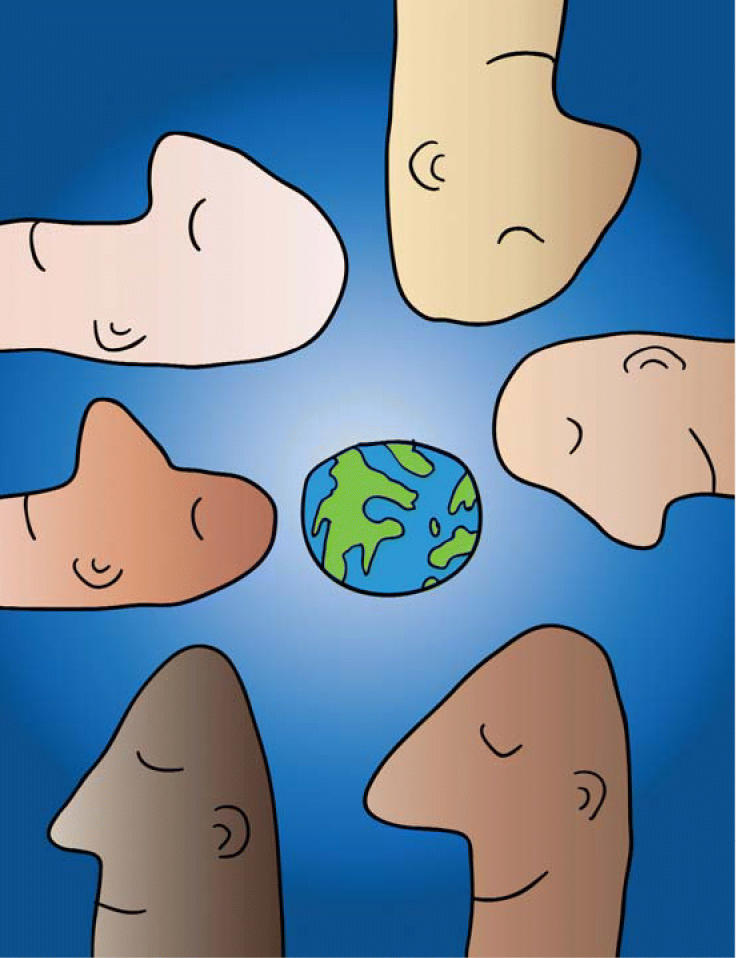# RTP Leaders Unite to Advance Environmental Health

**DOI:** 10.1289/ehp.114-a524

**Published:** 2006-09

**Authors:** Luz Claudio

When North Carolina’s Research Triangle Foundation provided 509 acres of land to the U.S. Surgeon General’s Office in 1967 as the site for the newly established Division of Environmental Health Sciences, the area was probably not foreseen as a hub for companies, institutions, and government agencies working on issues related to environmental health. Then, just two years later, the Division of Environmental Health Sciences was elevated to institute status to form the NIEHS. Since that time, the area now known as Research Triangle Park (RTP) has expanded into a nucleus of intellectual activity in environmental health sciences that includes the National Toxicology Program, the laboratories of the U.S. EPA, the CIIT Centers for Health Research, and environmental research programs at Duke University, the University of North Carolina–Chapel Hill, and North Carolina State University, among other institutions and nonprofit organizations.

These organizations are now taking advantage of a unique opportunity to solidify RTP’s reputation as the epicenter for environmental health science research in the United States by creating a forum for discussion and debate of the important public health issues related to environment and health. Prominent individuals in the RTP community—including former North Carolina governor James Hunt, former NIEHS director Kenneth Olden, and William Roper, chief executive officer of the North Carolina Health Care System and former head of the CDC—have been working to bring thought leaders together on these issues in a new initiative that has been dubbed the Research Triangle Environmental Health Collaborative.

The mission of the collaborative is to connect organizations and institutions; link research and policy; and join government, academia, industry, and public interest groups for the purpose of mutually considering, discussing, and debating the grand challenges in environmental health at the regional, national, and international levels. Says Olden, “When I came to the NIEHS many years ago, I realized the talent base we have here in RTP. The major environmental health research institutions are all here, the intellectual resources of the major research universities, and also the companies that have evolved around this. No place else in the world can boast this concentration of minds working on environmental public health issues. So we thought that it follows that if you can help to focus these talents in the areas where perhaps the most change can be effected, real progress might be made.”

## An Idea Made Real

According to David P. Brown, director of scientific research program development for Constella Group, a professional health consulting service that is facilitating the effort with support from the NIEHS, the collaborative has applied for nonprofit 501(c)(3) status. “Once created, the collaborative will provide those in government, academia, and the private sector with a neutral forum to host candid discussions and to provide advice on the most significant issues facing environmental health and related public policy,” says Brown.

“Constella is proud to work with the NIEHS and other organizations and be at the genesis of creating a forum to discuss exciting, ground-breaking issues in environmental health,” says Donald A. Holzworth, Constella Group’s chairman and CEO. “By articulating this vision for a public–private partnership focused on environmental health, we have a unique opportunity to participate in creating a global forum to build awareness and education around these critical issues.”

Olden chairs the collaborative’s Executive Committee, which currently comprises representatives from academia and the private sector. The other members include William Greenlee, president and CEO of the CIIT Centers for Health Research; David Hinton, Nicholas Professor of Environmental Quality at Duke University’s Nicholas School of the Environment and Earth Sciences; Ernest Hodgson, a professor of environmental and molecular toxicology at North Carolina State University; Rich Cohn, vice president of the Center for Health Research at Constella Group; and Edward Baker, director of the Institute for Public Health at the University of North Carolina–Chapel Hill.

Committee members hope that leaders of nonprofit organizations and public interest groups in RTP will join the collaborative as well.

## A First Step

The collaborative has already received $30,000 in initial funding from the NIEHS to host its first Environmental Health Summit, planned for the spring of 2007 in RTP. This meeting will bring together environmental and public health leaders from around the world to identify today’s foremost environmental health challenges. Participants, who may include former EPA administrators Carol Browner and William Reilly, will also seek to address these challenges from a solution-oriented perspective, emphasizing research needs, policy changes, education, and prevention/intervention programs.

The Environmental Health Summit is just one activity through which the collaborative plans to address important issues. The collaborative will help facilitate the goals of the NIEHS and the EPA by organizing meetings, helping to attract new scientists, and serving as a clearinghouse for environmental health science–related activities and events in the RTP area. The collaborative may also provide expert advice and review as well as a neutral forum for discussions on science and policy.

Other activities will include promoting RTP as a desirable scientific, research, and business destination in an effort to lure more environmental health science organizations to the area, as well as supporting the convergence of government, academia, and nonprofit interests to generate an attractive economic environment for business growth for private companies. The group will showcase academic institutions’ key environmental programs, including those supported by the federal government, and will facilitate interaction between academia, government, nonprofit foundations, public interest groups, and private organizations in a collegial environment. The collaborative also plans to facilitate multi-partner efforts in grant development to generate new research programs. Finally, the collaborative will serve as an interorganizational think tank comprising environmental, health, and policy leaders from associations, foundations, and nonprofit organizations.

For additional information about the collaborative, please contact David Brown at dabrown@constellagroup.com.

## Figures and Tables

**Figure:**